# Socio-economic position and subjective health and well-being among older people in Europe: a systematic narrative review

**DOI:** 10.1080/13607863.2015.1023766

**Published:** 2015-03-25

**Authors:** Sanna Read, Emily Grundy, Else Foverskov

**Affiliations:** ^a^ Department of Social Policy, London School of Economics and Political Science, London, United Kingdom

**Keywords:** health inequalities, socio-economic position, subjective health and well-being, older age, systematic narrative review

## Abstract

**Objectives:** Previous studies of older European populations have established that disability and morbidity vary with indicators of socio-economic position (SEP). We undertook a systematic narrative review of the literature to ascertain to what extent there is evidence of similar inequalities in the subjective health and well-being of older people in Europe.

**Method:** Relevant original research articles were searched for using Medline, Global Health, Embase, Social Policy and Practice, Cinahl, Web of Science and International Bibliography of the Social Sciences (IBSS). We included studies of SEP and indicators of subjective health and well-being (self-rated health; life satisfaction; quality of life) conducted since 1991 using population-based samples of older people in Europe and published 1995–2013.

**Results:** A total of 71 studies were identified. Poorer SEP was associated with poorer subjective health and well-being. Associations varied somewhat depending on the SEP measure and subjective health and well-being outcome used. Associations were weaker when social support and health-related behaviours were adjusted for suggesting that these factors mediate the relationship between SEP and subjective health and well-being. Associations tended to be weaker in the oldest age groups. The patterns of associations by gender were not consistent and tended to diminish after adjusting for indicators of health and life circumstances.

**Conclusion:** The results of this systematic narrative review of the literature demonstrate the importance of social influences on later life subjective health and well-being and indicate areas which need further investigation, such as more studies from Eastern Europe, more longitudinal studies and more research on the role of mediating factors.

## Introduction

Population ageing means that investigating and understanding the underlying determinants of health among older people is an important priority (Christensen, Doblhammer, Rau, & Vaupel, 2009; Doyle, McKee, Rechel, & Grundy, 2009), especially for Europe, the world region in which population ageing is most advanced. There is extensive evidence of considerable social inequalities in the health in older people in Europe but most of this evidence comes from studies, and reviews of these studies, which have focused on negative health outcomes such as mortality, morbidity and disability (Di Cesare et al., 2013; Huisman, Read, Towriss, Deeg, & Grundy, 2013; Pikhart et al., 2006,2012). However, it is also important to consider indicators of positive dimensions of health and well-being which may have a larger subjective component (Bowling, 1993). Although there are a number of reviews of aspects of subjective well-being in older populations (Hambleton, Keeling, & McKenzie, 2009; Netuveli & Blane, 2008; Ryff, 2014), socio-economic inequalities in these indicators have received less attention. To our knowledge, only one review has looked at socio-economic predictors of subjective well-being in older people (Pinquart & Sorensen, 2000). This review included studies carried out up to spring 1999 and did not consider studies using self-rated health and quality of life as outcomes, although these are widely used indicators of subjective health. In this paper, we report results from a systematic narrative review of studies published 1995–2013 on socio-economic inequalities in the subjective health and well-being of older Europeans focusing on studies which have investigated differentials in self-rated health, quality of life or life satisfaction. This review thus both updates and expands on the earlier one undertaken by Pinquart and Sorensen (2000).

We report results from studies using a number of indicators of socio-economic position (SEP), some of which relate to particular hypothesised mechanisms underlying associations between SEP and health. Indicators of material circumstances include income, wealth and housing tenure and related ecological indicators, notably area deprivation. These all refer to current circumstances – although clearly they reflect the accumulation of resources over the life course (Kaplan, Pamuk, Lynch, Cohen, & Balfour, 1996). Other indicators, namely education and also past occupation, relate to earlier points in the life course and, particularly in the case of education, may have greater salience for interpretations of linkages between SEP and health which emphasise behavioural and psychosocial pathways (Schrijvers, Stronks, van de Mheen, & Mackenbach, 1999). In their previous review, Pinquart and Sorensen (2000) found that an indicator of current SEP – current income – seemed more important than education for older people's subjective well-being.

The current review focuses on three outcome measures: self-rated health, quality of life and life satisfaction. Because these terms are only partly overlapping (Bowling, 2013), they were treated as separate outcomes in the analysis. Self-rated health is measured using a single-item question on perceived general health status which could include physiological, psychological and social dimensions (Miilunpalo, Vuori, Oja, Pasanen, & Urponen, 1997). Life satisfaction represents a judgemental component of subjective well-being, indicating the extent that life has meaning, goal and direction (Pavot & Diener, 2009). It can be measured using a global single-item or a scale covering different domains. Life satisfaction and well-being, as well as perceived health, are components of broader quality of life. Because quality of life represents the perceived ‘goodness’ of different dimensions of life, these measures typically include a range of items (Bowling, 2013). In the present review, we include both health-related (e.g. SF-36) and broader quality of life (e.g. CASP-19) measures.

We also assess the evidence that gender and age moderate associations between SEP and subjective health and well-being (i.e. whether associations differ by age group or gender). The previous review suggested that the association between SEP and subjective well-being was stronger in men than women, perhaps because men traditionally may be more career-orientated and SEP may play a more important role in defining their identity and influencing their well-being (Pinquart & Sorensen, 2000). The same review also found that inequalities in subjective well-being were smaller in the oldest age groups. In order to further explore mechanisms underlying associations between SEP and subjective health and well-being, we additionally consider whether studies suggest that health inequalities are mediated by health-related behaviours and social support (i.e. whether including indicators of these ‘explains’ inequalities). Previous studies in general populations indicate that health-related behaviours and social support are associated with both SEP and health and could be on the causal pathway between these two, suggesting potential mediation (Kawachi, Subramanian, & Almeida-Filho, 2002; Lantz et al., 2001; Vonneilich et al., 2012).

### Aim of the review

The questions addressed in this literature review are the following.

1. To what extent is there evidence from the literature of inequalities in the subjective health and well-being of older people in Europe by educational level and other indicators of SEP?
2. Is there evidence from the literature that health-related behaviour and social support potentially mediate associations between SEP and subjective health and well-being?
3. Is there evidence from the literature that associations between SEP and subjective health and well-being differ by age group or gender, i.e. whether there is SEP*age or SEP*gender interactive association with subjective health and well-being?

## Methods

### Definition of the terms used in the review

We investigated differentials in subjective health and well-being by educational level, income, wealth, financial assets (including indicators of wealth or assets such as home or car ownership), occupation-based social class measures and area-based indicators of deprivation. As indicated above, we included studies using self-rated health, life satisfaction and quality of life as measures of subjective health and well-being. The focus was on population-based studies of people aged 60+, including studies of a wider age range provided that separate results were presented for those aged 60 or older. We included studies focusing on particular ethnic groups but excluded those restricted to sub-groups defined by the use of a service or similar special characteristic (patients, people living in institutions, care givers, third age university students, etc.). The review focused on countries within Europe as geographically defined (see Table 1).

**Table 1.** Search words for the literature search on health inequalities.

Search limitation	Search words^1^
Subjective health and well-being	Subjective health, self-rated health, life satisfaction, quality of life, well-being
Inequalities	Inequality, disparity, education, socio-economic, wealth, income, financial assets, housing tenure, car owner, deprivation, occupational class, social class
Europe	Europe, Albania, Andorra, Austria, Belarus, Belgium, Bosnia, Herzegovina, Bulgaria, Croatia, Czech Republic, Denmark , Estonia, Finland , France, Germany, Greece, Hungary, Iceland, Ireland, Italy, Latvia, Lithuania, Luxembourg, Macedonia, Malta, Moldova, Monaco, Montenegro, Netherlands, Norway, Poland, Portugal, Romania, Russia, San Marino, Serbia, Slovakia, Slovenia, Spain, Sweden, Switzerland, Yugoslav Republic, Ukraine, United Kingdom
Age of population	age 60+, ageing, elderly, older people
Year of publication	1995–current (October 2013)
Type of publication	Article

^1^ Searches allowed terms with synonyms to be included. Difference in the US and English spelling and different alternatives for the search words were taken into account in the searches.

### Search strategy

A systematic literature search was undertaken of the following bibliographic databases: Medline, Global Health, Embase, Social Policy and Practice, Cinahl, Web of Science and International Bibliography of the Social Sciences (IBSS). Papers published as original research articles between 1995 and 2013 were included if they met our inclusion criteria. Searches were saved in Endnote, merged to a combined file and duplicate publications were deleted.

Key search terms were determined by the review question and the inclusion criteria. Search words used are shown in Table 1. Searches were carried out on the title and abstract of papers using search strings to select studies that were relevant in scope, population and type of publication. Papers were included in the review if they met the following criteria.

1. Geographical location: European region.
2. Population: people aged 60+ included in a general sample.
3. Scope: reporting socio-economic differences in subjective health and well-being (self-rated health, quality of life or life satisfaction).
4. Type of publication: journal article reporting an original study.
5. Date of publication: 1995–current (7/10/2013).
6. Date of data collection: 1991 onwards. For longitudinal studies, at least one follow-up, 1991 onwards. If the survey year was not reported, other sources were used to determine timing of data collections.
7. Language: any.

In the first step, titles and abstracts were screened and full reports obtained for studies appeared to meet the criteria or provided insufficient information to decide. Full reports were then reviewed using the same inclusion criteria.

### Full-text coding

The studies remaining after application of the criteria were coded to identify key elements relevant to the study questions.

1. Type of outcome measure: self-rated health, life satisfaction, quality of life, well-being.
2. Type of social determinant/s measured and investigated: education and SEP (income, wealth, financial assets, etc.).
3. Whether the study investigated and reported any intermediate effects of health-related behaviours or social support.
4. Whether the study investigated and reported the association between SEP and health outcomes by age or gender (SEP*age or SEP*gender interactions).

We also coded papers according to the methods they used.

1. Whether a longitudinal design was used
2. What covariates the study included: none; socio-demographic only; socio-demographic plus health status and/or functioning and/or social contacts, social support, social network and /or health behaviours.
3. Analysis method used: descriptive, bivariate, multivariable.
4. Response rate of study: under 50%, 50%–74%, 75% or higher.

At all steps of the screening (on title and abstract, full-text, and then coding), a sample of studies (about 10%) were screened by two authors (–SR and –EG). This was done to ensure consistency in the use of the inclusion criteria.

## Results

### Search results

**Figure 1.**
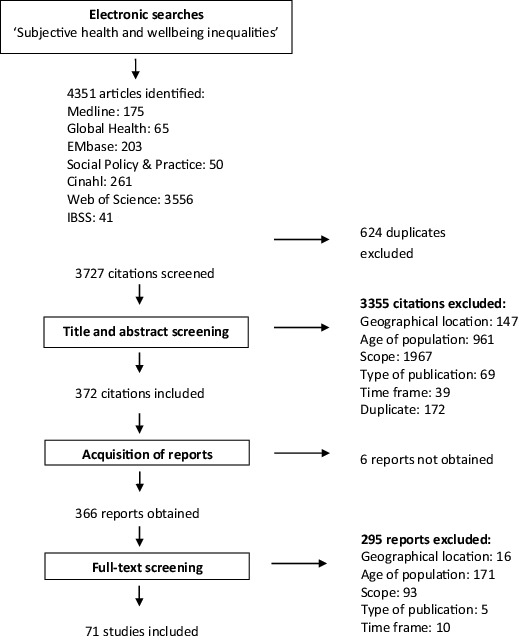
Results of the search and screening.

Figure 1 illustrates the process of filtering from searching and screening. A total of 4351 citations on inequalities in subjective health were identified. Duplicates (*n* = 624) were excluded after which 3727 citations were included for further screening. Titles and abstracts were screened using the inclusion criteria (described above). The majority of papers excluded at this stage (*n* = 1967) did not meet the inclusion criterion relating to scope of the study (i.e., they did not investigate socio-economic differences in subjective health and well-being). The second most common reason for exclusion was because the study did not include older people or did not report separate results for older people (*n* = 961).

Six papers were not obtained for full-text screening. These papers were published in small, national journals that could not be accessed via any of the 15 major libraries (in different countries) we tried. The abstracts of these papers in any case suggested that separate results for older people were not reported. Papers in languages other than English were translated for the purpose of the review by the authors or their collaborators who were native or fluent speakers of the language in question.

In total, 366 papers went through to full-text screening. Of these, 295 were excluded, most often because of the age of the population included or reported on (*n* = 171). After full-text screening, 71 reports were reviewed to answer the key review questions. Details of these studies are provided in the following sections.

### Study methodologies

Self-rated health (Table 2) was studied using a single-item question. For measuring quality of life (Table 3), in all cases, only a questionnaire was used, whereas for life satisfaction (Table 4) both single-item questions and questionnaires were used. The number of items and range and wording of the response scales varied between the measures. The full names of the scales and their contents can be found in Supplementary Tables 1 and 2. Only six studies (8%) examined associations longitudinally. Thirty-three papers (46%) used a large number of covariates including demographic covariates as well as measures of health, health-related behaviour, social support or other psycho-social factors. Fifteen papers (21%) included age and/or gender only. Four papers (6%) used no covariates or stratification in the models fitted.

**Table 2.** Characteristics of studies included, outcome measure: self-rated health.

Author(s), year	Survey year (waves)	Location	Sample – total	Sample –review	Age–total	Age – review	Inequality measure^1^	COV^2^	Stratified analysis^3^
Alwan et al. (2007)	2001(1)	Sheffield, UK	N/A	N/A	50+	65+	**Four area deprivation measures**	A	**Age**
Araujo, Ramos, and Lopes (2011)	2007(1)	Porto and Vinhais, Portugal	463	463	60+	60+	**Education**	–	–
Bambra et al. (2010)	2002–2006(1)	17 European countries	85,514	9938	15+	61+	**Education**	B	Sex
Christelis, Jappelli, Paccagnella, and Weber (2009)	2004(1)	11 European countries	9145	9145	65+	65+	**Net worth-income**	–	–
Connolly et al. (2010)	2001(1)	Northern Ireland	191,848	191,848	65+	65+	**Housing tenure, house value**	ABC	Sex, **age**
Dalstra et al. (2006)	1991–1999(1)	10 European countries	43,479	43,479	60–79	60–79	**Housing tenure, income, education**	ABC	Sex
Damian et al. (1999)	1994–1995(1)	Madrid, Spain	677	677	65+	65+	**Social class**	ABE	Sex, **age**
Enroth et al. (2013)	2010(1)	Tampere, Finland	1283	1283	90–107	90–107	**Occupation, education**	AB	Sex
Fernandez-Martinez et al. (2012)	2008(1)	Spain	1106	1106	60–96	60–96	Education, **financial position**	ABCE	–
Giron (2012)	2006(1)	Spain	7835	7835	65+	65+	**Education, income**, social class	ACDE	–
Gonzalo and Pasarin (2004)	1997–1999(1)	Spain	1072	1072	65+	65+	**Social class**	B	Sex
Grigoriev and Grigorieva (2011)^4^	1996–2007(1)	Belarus	43,732	3838	20+	60+	**Education, income**, living standards	ABCDE	–
Grundy and Holt (2000)	1994(1)	Great Britain	2247	2247	60–75	60–75	**Education,** income, **social class, housing tenure, life course unemployment**	ABC	**Sex**
Grundy and Sloggett (2003)	1993–1995(1)	England	8672	8672	65–84	65–84	**Education, income support, housing tenure**	ABCDF	**Sex**
Huijts et al. (2010)	2002–2006(1)	4 Nordic countries	17,801	3578	25+	65+	**Income**	BC	**Sex**
Huisman et al. (2003)	1994(1)	11 European countries	31,350	31,350	60+	60+	**Education, income**	AB	**Sex, age**
Karlsdotter et al. (2011)	2007(1)	Spain	6259	6259	65–80	65–80	**Education, income, four regional measures**	ABC	**Sex**
Knurowski et al. (2004)	1999–2001(1)	Krakow and Zagreb	814	814	65–85	65–85	**Education**	ABE	Sex
Knurowski et al. (2005)	1999–2001(1)	Krakow, Poland	528	528	65–85	65–85	**Education, occupation**, income, **homeowner**	AB	**Sex, age**
König et al. (2010)	2001–2003(1)	Six European countries	1659	1659	75+	75+	**Education**, income	ABCE	–
Lasheras et al. (2001)	N/A	Oviedo, Northern Spain	352	352	65–95	65–95	**Education**	ABC	**sex**
Malnar and Kurdija (2012)	2011(1)	Slovenia	1079	N/A	18+	60+	**Education**	–	–
Maniecka-Bryla, Drygas, Bryla, and Dziankowska-Zaborszczyk (2011)	N/A(1)	Łódź-Górna, Poland	768	768	65–74	65–74	**Education**	BCDE	–
McFadden et al. (2008)	1993–1997(1)	Norfolk, UK	22457	1382	39–79	65–79	**Occupational class**	AB	**Sex, age**
McMunn et al. (2009)	2002–2004(2)	England	6371	N/A	50+	65+	**Wealth**, income, **housing tenure**	AB	Sex, **age**
Melzer et al. (2000)	1991(1)	Four English cities	10,377	10,377	65+	65+	**Social class**	AB	Sex, age
Nummela et al. (2007)	2002(1)	Päijät-Häme county, Finland	2815	1548	52–76	62–76	**Education, adequacy of income, income**	ABC	Age
Orfila, Ferrer, Lamarca, and Alonso (2000)	1986–1994(2)	Barcelona, Spain	754	754	65+	65+	**Education**	ABCDE	–
Parker et al. (2013)	1968–2004(2)	Sweden	1131	1131	69–88	69–88	**Education, social class**	ABC	Sex
[Bibr cit0059]Perula de Torres et al. (1997)	1994(1)	Córdoba, Spain	1103	1103	60–94	60–94	**Education,** income, **adequacy of income**	ABC	–
Pirani and Salvini (2012)	2004–2005(1)	Italy	25,183	25,183	65+	65+	**Education, housing quality, economic resources**	ABCDEF	Age
Rueda and Artazcoz (2009)	2006(1)	Catalonia, Spain	2597	2597	65–85	65–85	**Education**, household resources	ABCF	Sex
Rueda et al. (2008)	2004(1)	10 European countries	9225	9225	65–85	65–85	**Education**, household income	ABC	Sex
Rueda (2012)	2006(1)	Four Spanish regions	1602	1602	65–85	65–85	**Socio-economic development of the region**	ABCF	**Sex**
Schöllgen, Huxhold, and Tesch-Roemer (2010)	2002(1)	Germany	2787	887	40–85	70–85	Education, income, **financial assets**	BC	–
Sulander et al. (2009)	1993–2003(1)	Finland	114,86	11,486	65–84	65–84	**Education**	AB	Sex
Sulander et al. (2012)	2008(1)	Helsinki, Finland	1395	1395	75–99	75–99	**Education, economic resources**	ABC	**Sex**, **age**
Tigani, Artemiadis, Alexopoulos, Chrousos, and Darviri[Bibr cit0078] (2012)	2007–2010(1)	Greece	400	400	100+	100+	Education, income, **financial problems**	BCDEF	–
Van Ourti (2003)	1994–1998(5)	Belgium	<26,000	<6000	15+	65+	**Current income**, permanent income	AB	--
von dem Knesebeck et al. (2003)	2000(1)	Germany	682	682	60+	60+	Education, **income, occupation,** assets, home ownership	ABC	Age
von dem Knesebeck et al. (2006)	2003(1)	22 European countries	36263	N/A	25+	61+	**Education**	AB	Sex, **age**
von dem Knesebeck (2005)	2000(1)	Germany	682	682	60+	60+	**Education, income, occupational status**	ABF	**--**
Vuorisalmi et al. (2008)	2000(1)	St Petersburg, Russia	1168	1168	60-89	60-89	**Occupational class**	ABE	–
[Bibr cit0088]Wroblewska (2002)	1996(1)	Poland	25,123	6524	15+	60+	**Education, financial position**	ACD	Age

^1^Socioeconomic position (SEP) measures in bold are the ones found to be significantly associated with self-rated health in final fully adjusted models.

^2^Covariates (COV) included in the analysis: A = age; B = gender; C = socio-demographic; D = health behaviour; E = other health status/disability; F = social support/contact.

^3^Stratified results shown for sex or age, text in bold indicates interaction sex*SEP or age*SEP on self-rated health.

^4^Only men included in the review because they fulfil the criteria for age (60+).

**Table 3.** Characteristics of studies included, outcome measure: quality of life.

Author(s), year	Survey year (waves)	Location	Sample – total	Sample – review	Age – total	Age – review	SEP measure^1^	Outcome measure	COV^2^	Stratified analysis^3^
Bowling and Stenner (2011)	2007–2008(1)	Great Britain	1276	1276	65+	65+	housing tenure	WHOQOL-OLD, CASP -19, OPQOL score	ABCEF	–
Bowling, Banister, Sutton, Evans, and Windsor (2002)	2000–2001(1)	Great Britain	999	999	65+	65+	Education, social class, income, housing tenure	Single item	ABCEF	–
Breeze et al. (2005)	1991--1999(1)	Great Britain	5581	5581	75+	75+	**Area deprivation, social class**	PGCMS+SIP score	ABCDE	–
Breeze et al. (2004)	N/A(1)	Great Britain	6298	6298	75+	75+	**Tenure status, social class**	PGCMS+SIP score	ABCDEF	–
Chandola et al. (2007)	1991–2004(5)	London, England	10308	N/A	50–74	60–74	**Occupational status**	SF-36: two comp.	ABC	**Age**
Cramm et al. (2013)	N/A(1)	Rotterdam, Netherlands	945	945	70+	70+	Education, income, home ownership	SPF-IL score	ABCF	–
de Belvis, Avolio, Sicuro et al. (2008)	1999–2000(1)	Italy	33744	33744	60+	60+	**Education, adequacy of income**	SF-12: two comp.	ABCDEF	–
de Belvis, Avolio, Spagnolo et al. (2008)	1999–2000(1)	Lazio region, Italy	1601	1601	60+	60+	**Education, adequacy of income**	SF-12: two comp.	ABCDEF	**--**
Eviö, Pekkarinen, Sintonen, Tiitinen, and Valimaki (2007)	2002(1)	Southern Finland	1663	1663	60–70	60–70	Education	15D score	ACE	–
Gilhooly et al. (2007)	N/A(1)	Scotland	145	145	70–91	70–91	Area deprivation	LEIPAD, QoL	ABDEF	–
Halleröd (2009)	2002–2003(1)	Sweden	3053	3053	66–99	66–99	Social class, **income**	Two latent comp.	ABC	–
Knurowski et al. (2004)	1999–2001(1)	Krakow and Zagreb	814	814	65–85	65–85	**Education**	Cantril's ladder	ABE	Sex, age
Knurowski et al. (2005)	1999–2001(1)	Krakow, Poland	528	528	65–85	65–85	**Education, occupation, income, home ownership**	Cantril's ladder	AB	**Sex, age**
König et al. (2010)	2001–2003(1)	Six European countries	1659	1659	75+	75+	**Education**, income	SF-12: 2 comp.	ABC	**--**
Laudisio et al. (2013)	2004(1)	Tuscania, Italy	356	356	75+	75+	Education, economic resources	HUI3 score	ABDE	Sex, age
Orfila et al. (2006)	1993--1994(1)	Barcelona, Spain	544	544	72+	72+	Education, social class	NHP: emotional reaction subscale	ABCDE	Sex
Pavlovic et al. (2010)	2006–2007(1)	Croatia	396	396	70–79	70–79	**Education**, **pension**	SF-36: 2 and 8 comp.	ABC	**--**
Regidor et al. (1999)	1996(1)	Spain	7823	1890	25+	65+	**Education**	SF-36: 8 comp.	B	**Sex**
Rodriguez-Blazquez et al. (2011)	2011(1)	Spain	1106	1106	60+	60+	**education**	PWI score	–	–
Sherman, Forsberg, Karp, and Tornkvist (2012)	2006(1)	Stockholm, Sweden	583	583	75	75	Education	HI score	BCE	–
Schmidt et al. (2012)	2008(1)	Germany	2222	614	25+	65+	**Index of SEP**	SF-12: two comp.	A	**Age**
Stenzelius et al. (2005)	N/A(1)	Southern Sweden	4277	4277	75+	75+	**Occupational status**	SF-12: two comp.	ABCE	–
von Heideken Wågert et al. (2005)	2000(1)	Umeå, Sweden	199	199	85+	85+	Education	PGCMS score	ABCEF	–
von dem Knesebeck, Wahrendorf, Hyde, and Siegrist (2007)	2004(1)	10 European countries	15080	N/A	50+	65+	**Education, income, home owner, car owner, net worth**	CASP-12 score	BC	Age

^1^Socioeconomic position (SEP) measures in bold are the ones found to be significantly associated with quality of life in final fully adjusted models.

^2^Covariates (COV) included in the analysis: A = age; B = gender; C = socio-demographic; D = health behaviour; E = other health status/disability; F = social support/contact.

^3^Stratified results shown for sex or age, text in bold indicates interaction sex*SEP or age*SEP on quality of life.

**Table 4.** Characteristics of studies included, outcome measure: life satisfaction.

Author(s), year	Survey year (waves)	Location	Sample – total	Sample – review	Age – total	Age – review	Inequality measure^1^	Outcome measure	COV^2^	Stratified analysis^3^
Bockerman, Johansson, and Saarni (2012)	2000–2001(1)	Finland	1928	1928	60+	60+	**Education**, **income**	Single item	ABCE	–
Dykstra and Wagner (2007)	1990-1993(1)	Amsterdam and Berlin	1177	1177	70+	70+	Change in social class over the work career	Single item	ABC	Sex
Enkvist, Ekstrom, and Elmstahl (2012)	2001–2004(2)	Five Swedish municipalities	681	681	78–93	78–93	Education, economical sufficiency	LSI-A score	ABCEF	–
Gaymu and Springer (2012)	2004(1)	10 European countries	13,550	13,550	60+	60+	**Education, income, home ownership**	Single item	ABCEF	**Sex**
Lucchetti et al. (2008)	2003–2004(1)	Southern Italy	304	304	75+	75+	**Education**, **occupation, economic resources**	LSI-A score	ABCEF	**--**
[Bibr cit0050]Melendez et al. (2009)	N/A	Valencia, Spain	181	181	65–92	65–92	Education, income	LSI-A score	ABCE	–
Schmidt et al. (2012)	2008(1)	Germany	2222	614	25+	65+	Index of SEP	SWLS score	A	Age

^1^Socioeconomic position (SEP) measures in bold are the ones found to be significantly associated with life satisfaction in final fully adjusted models.

^2^Covariates (COV) included in the analysis: A = age; B = gender; C = socio-demographic; D = health behaviour; E = other health status/disability; F = social support/contact.

^3^Stratified results shown for sex or age, text in bold indicates interaction sex*SEP or age*SEP on life satisfaction.

Most papers (86%) used multivariable analysis. A small proportion only examined bivariate associations (8%) or were wholly descriptive (6%). Two studies (3%) had response rates lower than 50%. Twenty-six studies (37%) reported response rates between 50% and 74% and 13 studies (18%) reported participation rate of 75% or higher. Twenty-four studies (34%) did not report response rates and six studies (8%), based on samples from different countries, reported participation rates varying from one country to another.

### SEP and subjective health and well-being outcomes

Of the studies included, 44 reported on self-rated health, 24 on quality of life and 7 on life satisfaction. Tables 2–4 summarize the results of these studies. Most of the studies reported an association between at least some of the SEP measures and subjective health and well-being with the most consistent associations reported for associations between SEP – regardless of the indicator used- and self-rated health. In total, 28 out of 32 (88%) studies showed an association between education and self-rated health, 11 out of 12 (92%) between occupational class and self-rated health, 8 out of 10 (80%) between assets/home ownership and self-rated health and all 7 (100%) between self-rated adequacy of income and self-rated health. There were only three studies on area deprivation all of which showed an association between deprivation and poorer self-rated health. The association between income and self-rated health was less clear. Of the 19 studies using income, 11 (58%) reported an association between income and self-rated health.

Compared with the results for self-rated health, a smaller proportion of the papers examining quality of life reported an association with SEP. Nine out of 16 (56%) studies found an association between education and quality of life, 3 out of 6 (50%) between income and quality of life, 5 out of 8 (63%) between occupational class and quality of life, and 5 out of 8 (63%) an association between assets/home ownership and quality of life. Two studies used self-rated adequacy of income as a measure, both of which reported an association with the outcome. Of the two studies using area deprivation, one (with a considerably larger sample size) showed an association between deprivation and poorer quality of life.

There were fewer studies on life satisfaction and again associations with indicators of SEP were less clear than in the studies which focussed on self-rated health. Of these, 2 out of 3 (67%) studies reported an association between income and life satisfaction, and 3 out of 5 (60%) between education and life satisfaction. One study using assets/homeownership reported an association. One study on occupational status and one study on self-reported adequacy of income found no association with life satisfaction, but another reported that occupation and economic resources were associated with life satisfaction. None of the studies included investigated associations between area deprivation and life satisfaction.

Within the domains of self-rated health, life satisfaction and quality of life, results also varied by measures used and between sub-scales. In one study, occupational status was associated with better comparative self-rated health, but not with global self-rated health (Vuorisalmi, Pietila, Pohjolainen, & Jylha, 2008). Several studies which used the SF-36 or similar instruments reported differences in associations between SEP and quality of life subscales. Generally the association between education and quality of life was stronger for physical subscales than for the mental subscales of the SF-36 (König et al., 2010; Pavlovic, Korajlija, Simic, Bobic, & Corovic, 2010; Regidor et al., 1999; Schmidt, Petermann, & Braehler, 2012) and latent physical compared to psychosocial factors (Halleröd, 2009). One study reported the opposite: self-assessed income was associated with the mental but not with the physical component of quality of life (de Belvis, Avolio, Spagnolo, et al., 2008). Another study also suggested that occupational status differences were larger for the mental than physical components of quality of life (Chandola, Ferrie, Sacker, & Marmot, 2007). Two studies found that the strength of the association between SEP and the two quality of life subscales was very similar (de Belvis, Avolio, Sicuro, et al., 2008; Stenzelius, Westergren, Thorneman, & Hallberg, 2005).

### Social support and health-related behaviour

There were only few papers which considered the possible role of social support or health-related behaviours in the association between SEP and subjective health and well-being. Adjusting for social support and health-related behaviours reduced the size of the association between subjective health and SEP in two British studies (Breeze et al., 2004,2005). Similarly, a Dutch study reported that after adjusting for social capital the association between income and quality of life disappeared (Cramm, van Dijk, & Nieboer, 2013). There were however a number of studies where the adjustment had only a small or no effect. In a study of Northern Ireland, the relationship between housing tenure/value and self-rated health did not change when social support and quality of the physical environment was adjusted for (Connolly, O'Reilly, & Rosato, 2010). Similarly among older Germans, social contacts and social support contributed very little to the association between SEP and self-rated health (von dem Knesebeck, 2005). In a Spanish study, a higher prevalence of poorer self-rated health among older people in less socioeconomically developed regions slightly reduced when social support was taken into account but differences between the less and more developed regions were still significant (Rueda, 2012).

### The association between SEP and subjective health and well-being by age

A number of studies indicated that the association between SEP and subjective health became weaker at older ages. This pattern was found in a large study including several countries (von dem Knesebeck, Verde, & Dragano, 2006), three studies from the UK (Alwan, Wilkinson, Birks, & Wright, 2007; Connolly et al., 2010; McMunn, Nazroo, & Breeze, 2009), and one study each from Finland (Sulander, Pohjolainen, & Karvinen, 2012 education only), Spain (Damian, Ruigomez, Pastor, & Martin-Moreno, 1999), Poland (Knurowski et al., 2005) and Germany (Schmidt et al., 2012 for the physical component of quality of life only). In a panel study from Belgium, the pattern of attrition and mortality over a five-year period suggested that mortality was concentrated among the lower income groups which may account for the weakening association between SEP and poorer self-rated health at older ages (Van Ourti, 2003).

Some studies, however, report no interaction with age. This was the case for a study based on analysis of the European Community Household Panel (Huisman, Kunst, & Mackenbach, 2003), two studies from each of Finland (Nummela, Sulander, Heinonen, & Uutela, 2007; Sulander et al., 2012 adequacy of income only), Germany (Schmidt et al., 2012 for the mental component of quality of life only; von dem Knesebeck, Luschen, Cockerham, & Siegrist, 2003), Poland (Knurowski et al., 2004; Wroblewska, 2002), Italy (Laudisio et al., 2013; Pirani & Salvini, 2012) and three from England (Chandola et al., 2007; McFadden et al., 2008; Melzer et al., 2000). Some studies had a smaller sample size for older age groups, whereas others used stratified sampling to achieve numbers of about the same size in different age groups; however this difference did not seem to be systematically associated with whether or not associations between SEP and outcomes varied between age groups.

### The association between SEP and subjective health and well-being by gender

The association between socio-economic factors and health by gender was examined and reported on in several studies, with some variations in findings depending on the SEP indicator considered, as well as other factors. The association between SEP and subjective health and well-being were found to be stronger in men than in women in one study from the UK (Grundy & Sloggett, 2003 for income support and housing tenure) and another from Spain (Regidor et al., 1999). The opposite result, stronger associations in women compared to men, was reported in two studies from the UK (Grundy & Holt, 2000; Grundy & Sloggett, 2003 for education) and one from Spain (Lasheras, Patterson, Casado, & Fernandez, 2001). A number of studies did not find gender interaction, including one study with 17 countries (Bambra, Netuveli, & Eikemo, 2010); three studies from the UK (Connolly et al., 2010; McMunn et al., 2009; Melzer et al., 2000), five from Spain (Damian et al., 1999; Gonzalo & Pasarin, 2004; Orfila et al., 2006; Rueda & Artazcoz, 2009; Rueda, Artazcoz, & Navarro, 2008); two from Finland (Enroth, Raitanen, Hervonen, & Jylha, 2013; Sulander, Rahkonen, Nummela, & Uutela, 2009); and one each from Sweden (Parker, Andel, Nilsen, & Kareholt, 2013), Italy (Laudisio et al., 2013), Poland (Knurowski et al., 2005 for quality of life only); Poland and Croatia (Knurowski et al., 2004) and the Netherlands and Germany (Dykstra & Wagner, 2007).

Studies from England (McFadden et al., 2008), Finland (Sulander et al., 2012) and Poland (Knurowski et al., 2005) also suggest that the interaction with gender may disappear when comparing men and women in older age groups. In a Spanish study, area measures of deprivation were more strongly associated with self-rated health in women than in men (Karlsdotter, Martin Martin, & del Amo Gonzalez, 2011). However, there were no gender interactions in the associations between personal SEP (education and income) and self-rated health.

Studies using data from several countries show very mixed results. For instance, analysis of European Social Survey data for 22 countries showed no clear pattern by gender in the association between education and self-rated health (von dem Knesebeck et al., 2006). Similarly, in a comparison of 10 countries (Dalstra, Kunst, Mackenbach, & Inequ, 2006), different regions in Spain (Rueda, 2012) and four Nordic countries (Huijts, Eikemo, & Skalicka, 2010), associations between SEP measures and self-rated health varied by gender but in different directions among the countries/regions studied, moreover confidence intervals were wide and no statistically significant differences between the genders could be seen. Another study using data for 10 countries from the Surveys of Retirement and Ageing in Europe showed that the results varied widely by gender depending on the SEP measure and whether older men and women were living alone or with a partner (Gaymu & Springer, 2012). Fluctuation in the gender interaction depending on country, age group and SEP measure was also reported in a study of 11 countries (Huisman et al., 2003). Sample size, use of covariates or participation rates were not systematically associated with whether gender interactions were found or not.

## Discussion

This systematic narrative review examined reported SEP inequalities in the subjective health and well-being of older people in Europe. Results indicate strong evidence of an association between SEP and the subjective health and well-being of older people, some of which may be mediated by health-related behaviour and social support. The papers reviewed covered Northern, Western and Southern European countries, but only a few from Eastern Europe where this topic has been investigated to a much lesser extent.

The studies used a wide range of indicators of SEP and also a range of subjective health and well-being outcomes. This meant that it was not possible to undertake meta-analysis and our review focused on describing similarities and differences in the results, and comparing the studies using several criteria relating to methodology. Despite the variation in measures, the results showed some general patterns. Socioeconomic inequalities were more evident in self-rated health than in quality of life and life satisfaction. Possibly this is because life satisfaction, representing the psychological dimension of well-being, may be less affected by external circumstances such as wealth (Aknin, 2009) and more by internal factors such as personality (Diener, Suh, Lucas & Smith, 1999). Quality of life measures include a number of psychological components which may make them more associated with internal factors. The studies that separated mental and physical dimensions (e.g. SF-36 and its shorter versions) indeed showed that the association between education and quality of life was more consistent for the physical subscale than for the mental subscale. However, the difference in the strength of associations between occupational status and the two subscales was less clear.

Although a previous review (Pinquart & Sorensen, 2000) suggested that measures of current circumstances, such as income, had a stronger association with subjective well-being than indicators of past circumstances, such as education, the present review suggests that this may depend on the outcome. Education was frequently associated with self-rated health but the association between education and quality of life or life satisfaction was less consistent. Income, in turn, was less frequently associated with self-rated health and quality of life than other SEP measures. It is important to note that Pinquart & Sorensen (2000) did not include studies using self-rated health and quality of life as outcomes in their review. The current review also highlights quite a consistent association between area deprivation and subjective health and well-being. Although area deprivation has received considerable attention in the health inequalities literature, previous studies have mostly treated the whole adult population as one group making it impossible to disentangle results for older age groups. The results from this review of the few relevant studies available suggest that more attention should be paid to this topic.

Adjusting for hypothesised intermediate factors of social support and health-related behaviours reduced the association between subjective health and well-being and SEP to some extent. The results of the present review demonstrate the importance of social influences on later life subjective health and well-being and suggest a need for further investigation of possible mediating factors so that the pathways from SEP to subjective well-being are clarified. The papers reviewed used stepwise regression to assess the contribution of the potential intermediate factors. These and some previous studies in adult populations (Kawachi et al., 2002; Lanzt et al., 2004; Vonneilich et al., 2012) show that health-related behaviours and social support are associated both with SEP and health and theoretically on the causal pathway linking the two (that is, SEP influences health-related behaviours and social support which in turn influence health). Because of this, controlling for them in models may result in an initially apparent association between SEP and health disappearing. However, the proper assessment of mediation requires the use of path models and longitudinal data. Longitudinal studies would make it possible to establish temporal sequences between SEP and subjective health and well-being and clarify any role of reverse causation as it is possible that some measures of SEP, for example perceived adequacy of income, might be influenced by poorer subjective well-being

In terms of age, associations tended to be weaker in the oldest age groups. This may reflect reduced statistical power in the generally smaller samples of very old people. Low numbers in the oldest age groups were reported in many studies, but a weakening association by age was also reported in some studies that had relatively large and equivalent numbers in different age groups suggesting that low power alone does not wholly account for the weakening association. Other explanations may be the decreased salience of factors related to working life for those in the oldest groups, the operation of various selection effects and the changing sensitivity of SEP measures over the life time (Bowling, 2004; Grundy & Holt, 2000). Sensitivity may also vary depending on gender, cohort and country. Gender interactions were not consistent and tended to diminish after adjusting for health and life circumstances. Fluctuation in results by gender was especially evident in studies including several countries which may indicate that gender effects are context specific. Small numbers of men in the oldest age groups in some studies and the related differential survival of men and women to advanced ages may also be relevant.

A challenge both conceptually and for analysis is that in many European countries the older population, especially of women, is less differentiated on key indicators of SEP, such as level of education, than mid-life groups. Additionally, indicators such as current income may reflect a complex combination of influences including lifetime accumulation of assets, policies on income support, and household composition. It might be useful in future studies to add accumulative measures of SEP which may perform better than measures of current or past position (see Stewart & Napoles-Springer, 2003). Adjusting for other SEP measures (or other covariates) may also change the results (Wilkinson & Pickett, 2006). Some of the papers included reported results from models fitted in steps so that the effect of adding each measure can be inferred, but there were also a number of papers where either only the fully adjusted findings or results for each measure separately were presented. The measurement of outcomes poses challenges as culture, age, gender and SEP may influence health expectations, and therefore the reporting of indicators of health and well-being. Measuring subjective health and well-being and using self-assessment of SEP may also correlate because of use of the same source of information.

Other limitations of this review were that the search was restricted to published journal articles, and excluded grey literature which might be relevant and very recent. The use of only peer-reviewed papers on the other hand is reflected in the generally high quality of the studies (such as use of advanced methods and higher participation rate). The review focuses on Europe and results may not be generalisable to other world regions. It is also important to note that a number of studies had to be excluded because even though they included older people, they reported results only for the total sample undifferentiated by age. It is important to report the results for age groups in future studies, especially as inequalities tend to differ by age. This requires attention to sampling designs so that studies include sufficient numbers in the oldest groups.

In summary, lower SEP was associated with poorer subjective health and well-being among older Europeans. Very few studies considered the role of health-related behaviours and social support, but those that did suggested that these factors may partially mediate associations between SEP and subjective well-being. The weaker associations found with increasing age may reflect reduced statistical power in generally smaller samples of very old people, decreased salience of factors related to working life for those in the oldest groups and operation of various selection effects. Gender differences were not clear and tended to disappear when other factors were adjusted. Very few studies included samples from Eastern Europe and most studies were cross-sectional. Further longitudinal research, especially from studies including Eastern European countries, is needed to see if the associations identified in this review apply also to Eastern Europe and to *change* in subjective well-being. Appropriate analysis of longitudinal data would also help to clarify the possible role of reverse causation and pathways whereby SEP influences subjective well-being, an important step needed to inform design and testing of possible interventions to reduce inequalities.

## Supplementary Material

Supplementary_table_1_and_2.docx
