# Supplementary material for: Socio-economic position and subjective health and well-being among older people in Europe: a systematic narrative review
Source: Aging Ment Health. 2015 Mar 25;20(5):529–42. doi: 10.1080/13607863.2015.1023766 (PMC4784497; doi:10.1080/13607863.2015.1023766)
Supplement: Supplementary_table_1_and_2.docx [file camh_a_1023766_sm3020.docx]

| Supplementary Table 1: Quality of life outcomes and abbreviations | | | | | |
| --- | --- | --- | --- | --- | --- |
| **Abbreviation** | **Outcome measure** | **Number of items** | **Subscales (number of items)** | **Response scale** | **References** |
| Cantril’s ladder | Cantril’s ladder | 1 | self-rating on a ladder from 0 ‘the worst life one can imagine’ to 10 ‘the best possible life one can imagine’ | scale from 0 to 10 | Knurowski et al., 2004  Knurowski et al., 2005 |
| CASP-19 | Control, Autonomy, Pleasure and Self-realisation measure of Quality of Life | 19 | control (4), autonomy (5), pleasure (5), self-realisation (5) | 4-point Likert scales, ‘Often’ to ‘Never | Bowling & Stenner, 2011 |
| CASP-12 | Short Version of the Control, Autonomy, Pleasure and Self-realisation measure of Quality of Life | 12 | control (3), autonomy (3), self-realisation (3), pleasure (3) | 4-point Likert scales, ‘Often’ to ‘Never’ | von dem Knesebeck et al., 2007 |
| 15D | 15-dimensional health related quality of life instrument | 15 | mobility (1), vision (1), hearing (1), breathing (1), sleeping (1), eating (1), speech (1), elimination (1), usual activities (1), mental function (1), discomfort and symptoms (1), depression (1), distress (1), vitality (1), sexual activity (1) | 5 ordinal levels, wording vary | Eviö et al., 2007 |
| HI score | Health Index Score | 9 | energy (1), mood (1), fatigue (1), loneliness (1), sleep (1), vertigo (1), bowel function (1), pain (1), mobility (1) | 4-point scale, ‘very poor’ to ‘very good’ | Sherman et al., 2012 |
| HUI3 score | Health Utilities Index, Mark 3 | 15 | vision, hearing, speech, ambulation, dexterity, emotion, cognition, pain | range of scales and wording vary | Laudisio et al., 2013 |
| 2 latent comp. | 2 latent components estimated with confirmative factor analysis | 16 | psychosocial (6), physical (10) | range of scales and wording vary | Halleröd, 2009 |
| LEIPAD | LEIPAD questionnaire | 29 | physical functioning (5), depression (4), self-care (6), cognition (5), social functioning (3) and life satisfaction (6) | range of scales and wording vary | Gilhooly et al., 2007 |
| NHP | The Nottingham Health Profile | 38 | physical abilities (8), pain (8), sleep(5), social isolation (5), emotional reactions (9), and energy level (3) | yes/no | Orfila et al., 2006^b^ |
| OPQOL | Older People’s Quality of Life questionnaire | 35 | life overall (4), health (4), social relationships and participation (8), independence, control over life, freedom (5), area: home and neighbourhood (4), psychological and emotional well-being (4), financial circumstances (4), religion/culture (2) | 5-point Likert scales, ‘Strongly agree’ to ‘Strongly disagree’ | Bowling & Stenner, 2011 |
| PGCMS | Philadelphia Geriatric Center Morale Scale | 17 | agitation (6), attitude towards own aging (5), dissatisfaction (6) | 2 response options, vary in their wording | Breeze et al., 2005  Breeze et al., 2004  von Heidekan Wågert et al. 2005 |

| Supplementary Table 1 continued | | | | | |
| --- | --- | --- | --- | --- | --- |
| **Abbreviation** | **Outcome measure** | **Number of items** | **Subscales (number of items)** | **Response scale** | **References** |
| PWI | Personal Wellbeing Index | 7 | standard of living (1), health (1), achievements in life (1), personal relationships (1), safety (1), community-connectedness (1), future security (1) | 10-point Likert scales, ‘Completely dissatisfied’ to  ‘Completely satisfied’ | Rodriguez-Blazquez et al., 2011 |
| QoL | The Delighted-Terrible Faces scale | 1 | respondents indicate which of the seven faces varying in degree of smiling or scowling best represented their current quality of life | scale from 0 to 6 | Gilhooly et al., 2007 |
| SF-36 | Short-Form-36 Health Survey | 35^a^ | physical function (10), role limitations due to physical problems (4), bodily pain (2), general health perception (5), general mental health (5), role limitations due to emotional problems (3), energy/vitality (4), social functioning (2)  - can be summarised into a physical and mental health component | range of scales and wording vary | Chandola et al., 2007  Pavlovic et al., 2010  Regidor et al., 1999 |
| SF-12 | Short-Form-12 Health Survey | 12 | physical function (2), role limitations due to physical problems (2), bodily pain (1), general health perception (1), general mental health (2), role limitations due to emotional problems (2), energy/vitality (1), social functioning (1)  - can be summarised into a physical and mental health component | range of scales and wording vary | de Belvis et al., 2008a  de Belvis et al., 2008b  König et al., 2010  Schmidt et al., 2012  Stenzelius et al., 2005 |
| Single item | Global Quality of Life Rating | 1 | overall rating of quality of live | 7-point Likert scales, ‘So good it could not be better’ to ‘So bad it could not be worse’ | Bowling et al., 2002 |
| SIP | Sickness Impact Profile | 63 | home management (10), mobility (10), self-care (23), social interaction (20) | yes/no | Breeze et al., 2005  Breeze et al., 2004 |
| SPF-IL | Social Production Function Instrument for the Level of Well-being | 15 | comfort, stimulation, behavioural confirmation, affection, status | 4-point scale, ‘never’ to ‘always’ | Cramm et al., 2013 |
| WHOQOL-OLD | World Health Organizations Quality of Life measure for older adults | 24 | sensory abilities (4), autonomy (4), past present and future activities (4), social participation (4), death and dying (4), and intimacy (4) | 5-point Likert scales, vary in their wording | Bowling & Stenner, 2011 |

a: In addition the SF-36 has a question that covers change in health status over the past year, which is not counted in scoring the eight dimensions.

b: We only include results for the emotional reaction subscale in the review.

| Supplementary Table 2: Life satisfaction outcomes and abbreviations | | | | | |
| --- | --- | --- | --- | --- | --- |
| **Abbreviation** | **Outcome measure** | **Number of items** | **Subscales** | **Response scale** | **References** |
| LSI-A score | Life Satisfaction Index A | 20 | zest, resolution and fortitude, congruence between desired and achieved goals, positive self-concept, mood tone | scores from 0 to 2, ‘disagree’, ‘doubtful’ and ‘agree‘ | Enkvist et al., 2012  Meléndez et al., 2009  Lucchetti et al., 2008 |
| single item |  | 1 | respondents are asked, all things considered, how satisfied they have been with their life as a whole during the past 30 days | scale from 0 to 10 | Bockerman et al., 2012 |
| single item |  | 1 | respondents are asked how satisfied they are with their life in general | scale from 1 to 5, ‘very dissatisfied’ to ‘very satisfied’ | Dykstra & Wagner, 2007 |
| single item |  | 1 | respondents are asked if they are satisfied with their life in general | scale from 1 to 4, very satisfied to ‘very dissatisfied’ | Gaymu & Springer, 2012 |
| SWLS score | Satisfaction with Life Scale | 5 | in most ways my life is close to my ideal, the conditions of my life are excellent, I am satisfied with my life, so far I have gotten the important things I want in life, if I could live my life over I would change almost nothing | scales from 1 to 7, ‘strongly disagree’ to ‘strongly agree’ | Schmidt et al., 2012 |
